# Evaluation of the predictive values of elevated serum l-homoarginine and dimethylarginines in preeclampsia

**DOI:** 10.1007/s00726-022-03177-x

**Published:** 2022-06-26

**Authors:** Xiangmei Yuan, Leiming Cai, Fengmei Hu, Li Xie, Xiong Chen, Jingjing Wu, Qian Li

**Affiliations:** 1 Department of Laboratory Medicine, Wusong Branch, Zhongshan Hospital, Fudan University, Shanghai, 200940 China; 2 Shanghai AB Sciex Analytical Instrument Trading Co., Ltd., Shanghai, 200050 China; 3 Department of Gynecology and Obstetrics, Wusong Branch, Zhongshan Hospital, Fudan University, Shanghai, 200940 China

**Keywords:** l-Homoarginine, Dimethylarginine, Preeclampsia, Liquid chromatography–mass spectrometry, Prediction

## Abstract

l-homoarginine (hARG) is involved in nitric oxide biosynthesis, but its role and concentration in preeclampsia (PE) have not been fully revealed. The purpose of this study was to develop and validate a feasible clinical assay to quantify serum hARG, arginine (ARG), asymmetric (ADMA) and symmetric dimethylarginines (SDMA) levels by LC–MS/MS and investigate their differences at different stages of pregnancy with or without preeclampsia. Serum samples were collected from 84 pregnant women without complications (controls), 84 with mild preeclampsia (MPE), and 81 with severe preeclampsia (SPE) at various gestation stages (before the 20th week, during the 20th–28th week or after the 28th week of gestation). No significant difference in ARG levels was observed between PE and controls at any stage (*P* > 0.05). The serum hARG levels and hARG/ADMA ratios of MPE before the 20th week were higher than those of controls (*P* < 0.001). ADMA levels of MPE were higher than those of controls during the 20th–28th week (*P* < 0.01). SDMA levels of SPE were higher than those of MPE (*P* < 0.01) and controls (*P* < 0.05) after the 28th week. Elevated serum hARG before the 20th week was identified as an independent predictor for PE (OR = 1.478, 95% CI 1.120–1.950). ROC curve analysis showed serum hARG before the 20th week had a good potential to predict MPE (AUC = 0.875, 95% CI 0.759–0.948). In conclusion, our study indicated that elevated serum hARG and dimethylarginine levels detected by LC–MS/MS might serve as potential biomarkers for the early prediction of PE.

**Supplementary Information:**

The online version contains supplementary material available at 10.1007/s00726-022-03177-x.

## Introduction

Preeclampsia (PE) is a common multi-system hypertensive disorder of pregnancy that seriously affects maternal and neonatal morbidity and mortality worldwide. It is extremely important to identify biomarkers for early prediction of PE to enable early assessment of maternal risk before the onset of clinical symptoms and thus early intervention to reduce the mortality of PE (Rana et al. 2019). The pathogenesis of PE is complex and involves multiple pathophysiological factors. It seems difficult to predict the disease accurately using a certain single biomarker. Therefore, it is necessary to find more potential biomarkers of PE and evaluate various panels of biomarkers in the early prediction of PE (Grill et al. 2009).

Hypoxia, endothelial cell dysfunction, and aseptic systemic inflammation are thought to play a critical role in the pathogenesis of PE (Rana et al. 2019). Nitric oxide (NO) is an essential biological messenger molecule and effector molecule that regulates blood vessel tension, participates in nerve signal transmission, and mediates inflammation and immune response. The reduction of NO synthesis or utilization plays a vital role in the pathogenesis of PE (Khalil et al. 2015). NO is produced by nitric oxide synthase (NOS) with arginine (ARG) as substrate. ADMA is a nitric oxide synthase inhibitor that inhibits NO synthesis and is associated with impaired endothelial function and uterine artery blood flow disorder (Dymara-Konopka and Laskowska 2019). Meta-analysis of study data revealed that the significantly elevated ADMA concentrations in peripheral blood of PE patients were the main reason for the decreased bioavailability of NO in PE patients (Nemeth et al. 2018; Demir et al. 2012; Tashie et al. 2020). Elevated SDMA is an early marker of renal impairment. SDMA was thought to be better at reflecting renal function damage than estimating glomerular filtration rate by calculating the creatinine equation (Schwedhelm and Boeger 2011). Some studies suggested that the ratio of ARG/ADMA could be used as an indicator of change in NO synthase activity related to PE (Cardounel et al. 2007; Tsikas et al. 2000; Sandrim et al. 2010; Speer et al. 2008; Braekke et al. 2009). As a homolog of ARG, hARG is also a substrate for NO synthesis (Hecker et al. 1991). The serum hARG could replace ARG to synthesize NO to dilate blood vessels and inhibit platelet aggregation (Guenes et al. 2017). Valtonen et al. found the importance of hARG in pregnancy adaptation, and hARG may play a direct role in NO upregulation and participate in NO-related function as a NO precursor (Valtonen et al. 2008). Tomaschitz et al. found that low hARG levels were associated with decreased renal function and adverse cardiovascular outcomes (Tomaschitz et al. 2014).

Studies concerning ARG levels in PE showed contradictory results. Some studies reported increased ARG levels in PE, some reported ARG decrease in PE, and others reported no significant differences (Tashie et al. 2020; Braekke et al. 2009; Kim et al. 2006; Mao et al. 2010). Differences of analysis techniques in those studies might be responsible for the inconsistencies. LC–MS/MS is considered as “gold standard” for measuring these small molecular biomarkers with high accuracy, specificity, and sensitivity. In this study, we established a LC–MS/MS method for simultaneous and accurate determination of serum hARG, ARG, ADMA and SDMA concentrations and further verified the methodology. Based on this LC–MS/MS detection method, the clinical significance of serum hARG, ARG, ADMA and SDMA for early prediction of PE was assessed.

## Materials and methods

### Study population

This study was approved by the ethics committee of Wusong Branch, Zhongshan Hospital, Fudan University. In total, 165 patients clinically diagnosed with PE and 84 pregnant women without any pregnancy-related diseases (controls) who were delivered in the hospital from June 2014 to December 2018 were included in this study. According to the International Society of the Study of Pregnancy (ISSHP), PE is defined as newly developed hypertension after the 20th week of pregnancy (systolic blood pressure (SBP) ≥ 140 mmHg and/or diastolic blood pressure (DBP) ≥ 90 mmHg, on two occasions at least 4 h apart), and at least one of the following emerging symptoms: proteinuria ≥ 300 mg/24 h, or random proteinuria+ , or new terminal organ damage (thrombocytopenia, liver function impairment, renal function impairment, pulmonary edema, new central nervous system abnormalities, or visual impairment). Patients with PE were further divided into two subgroups, mild (MPE) or severe PE (SPE), based on symptom severity. SPE was severe hypertension (SBP ≥ 160 mmHg or DBP ≥ 110 mmHg, on two occasions at least 4 h apart), or proteinuria ≥ 300 mg/24 h, or random protein 3+ , or accompanied by terminal organ damage (Khalil et al. 2013). There were 84 samples from controls, including 28 samples at each pregnancy stage (before the 20th week, 20th–28th week, and after the 28th week). Eighty-four patients with MPE were collected, including 28 at each of the three stages, respectively. Eighty-one samples from SPE were obtained, including 26 before the 20th week, 28 in the 20th–28th week, and 27 after the 28th week. All subjects were singleton pregnancies without specific medication prior to blood collection. Pregnancy infection, diabetes, essential hypertension, cardiovascular disease, liver disease, kidney disease and other vital organ diseases were excluded. The clinical data of controls and PE women were comparable (except primiparous proportion, SBP and DBP), as shown in Table 1. All subjects including healthy pregnant woman received regular antenatal examination until delivery. Serum was collected from the pregnant women after at least 8 h of fasting and stored at − 80 °C until used. All the included samples were collected before clinical PE diagnosis.

**Table 1:** Clinical characteristics of the included subjects

Characteristics	Controls	MPE	SPE	*P* value
**Before the 20th week**				
Primiparous proportion	25% (7/28)	71% (20/28)	50% (14/26)	0.002
Age (years)	30.4 ± 5.1	27.1 ± 4.6	28.8 ± 5.7	0.061
BMI (kg/ m2)	22.0 ± 2.7	22.7 ± 3.3	23.4 ± 4.8	0.405
Diagnosis week of PE	**/**	35.7 ± 4.8	34.3 ± 6.2	0.337
GA at sampling (weeks)	14.6 ± 2.3	14.8 ± 2.2	15.0 ± 2.5	0.785
SBP (mmHg)	118.0 ± 7.7	115.2 ± 8.4	118.8 ± 10.6	0.287
DBP (mmHg)	74.3 ± 6.7	72.7 ± 6.1	72.7 ± 10.9	0.715
GFR [mL/(min*1.73m^2^)]	119.3 ± 8.3	123.6 ± 14.0	116.2 ± 16.1	0.121
**During the 20th–28th week**				
Primiparous proportion	(35%) 10/28	(68%) 19/28	(39%) 11/28	0.031
Age (years)	28.6 ± 4.3	29.0 ± 5.6	28.3 ± 4.5	0.844
BMI (kg/ m2)	22.5 ± 3.8	24.5 ± 5.2	23.5 ± 5.1	0.329
Diagnosis week of PE	**/**	36.5 ± 3.4	34.7 ± 6.2	0.184
GA at sampling (weeks)	24.4 ± 1.5	23.2 ± 2.9	23.7 ± 2.7	0.407
SBP (mmHg)	117.8 ± 8.0	128.1 ± 7.9	133.6 ± 10.1	< 0.001
DBP (mmHg)	75.5 ± 9.6	81.4 ± 7.2	83.3 ± 10.5	0.006
GFR [mL/(min*1.73m^2^)]	121.0 ± 16.0	110.7 ± 23.6	111.8 ± 28.3	0.194
**After the 28th week**				
Primiparous proportion	(46%) 13/28	(71%) 20/28	(44%)12/27	0.079
Age (years)	28.9 ± 3.7	29.8 ± 5.1	29.8 ± 8.8	0.839
BMI (kg/ m2)	22.0 ± 3.6	23.5 ± 3.8	22.9 ± 5.2	0.387
Diagnosis week of PE	**/**	33.6 ± 6.9	36.3 ± 3.1	0.077
GA at sampling (weeks)	36.2 ± 2.7	35.3 ± 2.4	34.7 ± 2.3	0.081
SBP (mmHg)	116.0 ± 9.2	141.3 ± 7.7	146.7 ± 12.5	< 0.001
DBP (mmHg)	74.1 ± 9.5	91.4 ± 7.9	95.7 ± 9.9	< 0.001
GFR [mL/(min*1.73m^2^)]	108.8 ± 14.1	110.5 ± 13.6	103.4 ± 20.3	0.245

*GA* gestational age; *SBP* systolic blood pressure; *DBP* diastolic blood pressure; *GFR* glomerular filtration rate

### Chemicals and reagents

Methanol and acetonitrile (HPLC grade) were purchased from Merck KGaA Company, Germany. Formate and ammonium formate (both LCMS grade) were purchased from Anaqua^™^ Chemicals Supply Company, USA. Deionized water was made by a Milli-Q system (Millipore, MA, USA). The pooled serum came from a mix of healthy human serum. The chromatographic column was TSK-GEL Amide-80, 2.0 × 150 mm I.D, 3.0 µm. Standards, including ARG, hARG, ADMA, SDMA, monomethylarginine, trimethyllysine, and acetyllysine were purchased from Sigma-Aldrich (St. Louis, MO, USA). The internal standards (IS), including ARG-^15^N4, hARG-d4, ADMA-d6, and SDMA-d6, were purchased from TRC (Toronto, ONT, Canada) and Cambridge Isotope Laboratories (Andover, MA, USA).

### Calibrators, quality control samples, and internal standards solutions

The precise amount of each standard was weighed and dissolved in methanol–water (1:4, V/V). The concentrations of ARG, hARG, ADMA and SDMA stock solution were 129,713 µmol/L, 50,470 µmol/L, 842 µmol/L and 337 µmol/L, respectively. The stock solution were stored at – 20 ℃. Dilute the stock solutions with methanol–water (1:1, v/v) and mix to obtain the working solutions containing all analytes of the seven-point calibrators (ARG: 10, 20,40, 80, 200, 400, 500 µmol/L; hARG: 0.2, 0.4, 1, 4, 8, 16, 20 µmol/L; ADMA: 0.1, 0.2, 0.4, 0.8, 2, 4, 5 µmol/L; SDMA: 0.1, 0.2, 0.5, 2, 4, 8, 10 µmol/L).

The stock solutions were diluted with methanol–water (1:1, v/v) and mixed to obtain a tenfold quality control working solution. The quality control samples were obtained by mixing the tenfold working solution with the pooled serum (1:9, V/V). As a result, the following spiked analytes for the quality control samples were achieved: high-level QC (QC-H) (375 µmol/L ARG, 15 µmol/L hARG, 3.75 µmol/L ADMA, 7.5 µmol/L SDMA), medium-level QC (QC-M) (75 µmol/L ARG, 3 µmol/L hARG, 0.75 µmol/L ADMA, 1.5 µmol/L SDMA) and low-level QC (QC-L) (30 µmol/L ARG, 0.6 µmol/L hARG, 0.3 µmol/L ADMA, 0.3 µmol/L SDMA). The quality control samples followed the testing of each batch of samples to ensure the accuracy of the results of each batch.

The appropriate amount of each internal standard was accurately weighed, dissolved in methanol–water (1:4, v/v), and the concentrations of ARG-^15^N4, hARG-d4, ADMA-d6, and SDMA-d6 stock solution were 457 µmol/L, 3526 µmol/L, 854 µmol/L, and 470 µmol/L, respectively. The IS stock solutions were further diluted with methanol–water (1:1, v/v) and mixed to obtain the IS working solution of the following concentrations: 5 µmol/L ARG-^15^N4_,_ 5 µmol/L hARG-d4, 2 µmol/L ADMA-d6, and 2 µmol/L SDMA-d6.

### Sample pretreatment

50.0 µL of the sample (calibrator working solution or quality control samples or serum samples) was mixed with 50.0 µL of the IS working solution and 100 µL acetonitrile. The mixture was vortexed and centrifuged at 14,000 rpm at 4 °C for 5 min. Then, 100 µL of the supernatant was diluted with 100 µL of 2% formic acid in methanol–water (1:1, v/v) and vortexed. Finally, 100 µL supernatant was taken for LC–MS/MS analysis after centrifugation at 4 °C at 14,000 rpm for 5 min.

### LC–MS/MS conditions

The LC–MS/MS analysis was performed using AB SCIEX Quad^™^ 4500MD and Shimadzu Jasper^™^ HPLC. Mobile phase A is an aqueous solution of 10 mM ammonium formate containing 0.1% formic acid, and mobile phase B is a 95% acetonitrile-aqueous solution of 10 mM ammonium formate containing 0.1% formic acid. The chromatography gradient (%B) was set as follows 90–75%B for 0–7 min, 75%B for 7–9 min, 75–90%B for 9–9.1 min, 90%B for 9.1–15 min. Gradient elution was carried out at a flow rate of 0.65 mL/min. The column temperature was maintained at 40 °C. The electrospray ionization-mass spectrometry detected the analytes with multiple reaction monitoring (MRM) positive ionization mode (ESI+). The optimized mass spectrometry parameters were as follows: vaporizer temperature, 500 °C; Gas1, 50 psi; Gas2, 55 psi; Curtain Gas, 30.0 psi; ion spray voltage, 5500 V. The compound parameters optimized for each analyte were listed in Table 2.

**Table 2:** The compound parameters of each analyte and corresponding internal standard using LC–MS/MS

Compound	Role	Q1, *m*/*z*	Q3, *m*/*z*	DP, volts	CE, volts	CXP, volts
ARG	Analyte	175.1	116.1	74	25	10
hARG	Analyte	189.1	84.1	80	30	11
SDMA	Analyte	203.1	172.1	70	18	12
ADMA	Analyte	203.1	46.1	80	38	6
ARG-^15^N4	IS	179.2	71.2	91	27	10
hARG-d4	IS	193.1	88.1	90	30	12
SDMA-d6	IS	209.1	175.1	80	19	16
ADMA-d7	IS	209.2	52.0	90	27	10

*DP* declustering potential; *CE* collision energy; *CXP* collision cell exit potential; *IS* internal standard; *Q1* parent ion; *Q3* product ions

### Method validation

To ensure the accuracy and reliability of the test results using this LC–MS/MS method, linearity, accuracy, precision (intra- assay /inter-assay), recovery, trueness, matrix effect (absolute and relative matrix effect), and stability were validated following Chinese Guidance for Liquid Chromatography and Mass Spectrometry Clinical Application and C62–A from Clinical and Laboratory Standards Institute (CLSI) for bioanalytical method validation.

### Linearity, accuracy and precision

Three consecutive batches of linear validation were performed. Taking the concentration of the analyte as the x-coordinate and the ratio of the peak area of the analyte to the internal standard as the y-coordinate, the weighted ($W = 1/{x}^{2}$) least-squares method was used for linear regression to obtain the standard curve. The acceptance criteria were the relative deviation of all points < 15.0%, except the lower limit of quantitation < 20%, and the correlation coefficient of calibration curve *r* > 0.990. The accuracy was assessed by the recovery experiment of quality control samples. Low, medium, and high-level quality control samples were tested five times in each run, and three runs were completed in at least two days. The ratio of test value and theoretical value of quality control samples were calculated as accuracy. The precision was evaluated by intra-assay CV (*n* = 5) and inter-assay CV (*n* = 15 for each analyte).

### Recovery

Low and high levels of analytes were added to the pooled serum to prepare sample A. Three copies of each sample were prepared in parallel and pre-treated. The pooled serum was pre-treated without adding internal standards, then sample B was prepared by adding the corresponding internal standards and analytes to the post-extraction solution. The extraction recovery was equal to the peak area of sample A divided by the peak area of sample B to evaluate whether the extraction recovery of internal standard and analyte in the pretreatment is consistent and reproducible.

### Trueness

Serum samples from six people were divided into three parts for each sample, two of which were added with analytes to prepare the low and high-level-spiked samples. After pretreatment, samples were injected for analysis, and the accompanying standard curve determined the concentration of each analyte in each sample. The trueness was calculated as the following formula: Trueness (%) = the tested concentration of low or high-level-spiked samples/(the tested concentration of no analytes spiked samples + the theoretical concentration of the analyte spiked) ×100%, to ensure that the detection was efficient and accuracy in different human’s substrates.

### Matrix effect (absolute and relative matrix effect)

Three serum samples from different people were taken and pre-treated without internal standards. Sample C was prepared by adding Internal standards and analytes to the extracted matrix. The same internal standard and analyte were spiked to the pure solvent to obtain Sample D. Sample E was prepared by adding Internal standards to the extracted matrix. The peak areas of analytes and internal standards in each sample were calculated, respectively. The matrix factor was calculated according to the following formula: absolute matrix effect = [analyte peak area of C-analyte peak area of E)/analyte peak area of D] × 100%. IS-normalized matrix factor = (analyte peak area/IS peak area of C-analyte peak area/IS peak area of E)/(analyte peak area/IS peak area of D) × 100%.

Sample F was prepared by adding analytes to the alternative substrate (50% methanol). Sample G was from 6 different people. Each sample G and sample F were mixed in 1:1 (v: v) to obtain sample H. Relative matrix effect = (ratio of analyte peak area/IS peak area of H)/(ratio of analyte peak area/IS peak area of F × 0.5 + ratio of analyte peak area/IS peak area of G × 0.5) × 100%

### Stability

Serum samples from different people were mixed and divided into three groups. One group was treated immediately, which is T0; One group was left at room temperature for five hours before pretreatment; One group was placed at – 80 ℃ to investigate the freeze–thaw stability. The relative deviation was calculated by comparing the concentration of each group with the concentration of T0.

### Statistical analysis

Statistical analyses were performed using SPSS 20.0 and MedCalc Version 15.2.2. The Shapiro–Wilk test was used to determine the normality of the data. For variables with skewed distribution, the data were expressed as the median and quartile interval (IQR 25–75%). Using Kruskal–Wallis analysis and the Mann–Whitney *U* test with Bonferroni post-hoc correction, multiple comparisons were performed. Logistic regression analysis was used to analyze factors related to PE. The receiver operating characteristic (ROC) curve analyses were performed with GraphPad Prism Version 8.0. The area under the curve (AUC) was used to evaluate the discriminating ability of serum biomarkers to distinguish the pregnancy with PE from normal pregnant women. Multiple ROC curves were compared using the DeLong method (DeLong et al. 1988), and *P* < 0.05 was considered statistically significant (Delong et al. 1988).

## Results

### Method validation summary

**Fig. 1:**
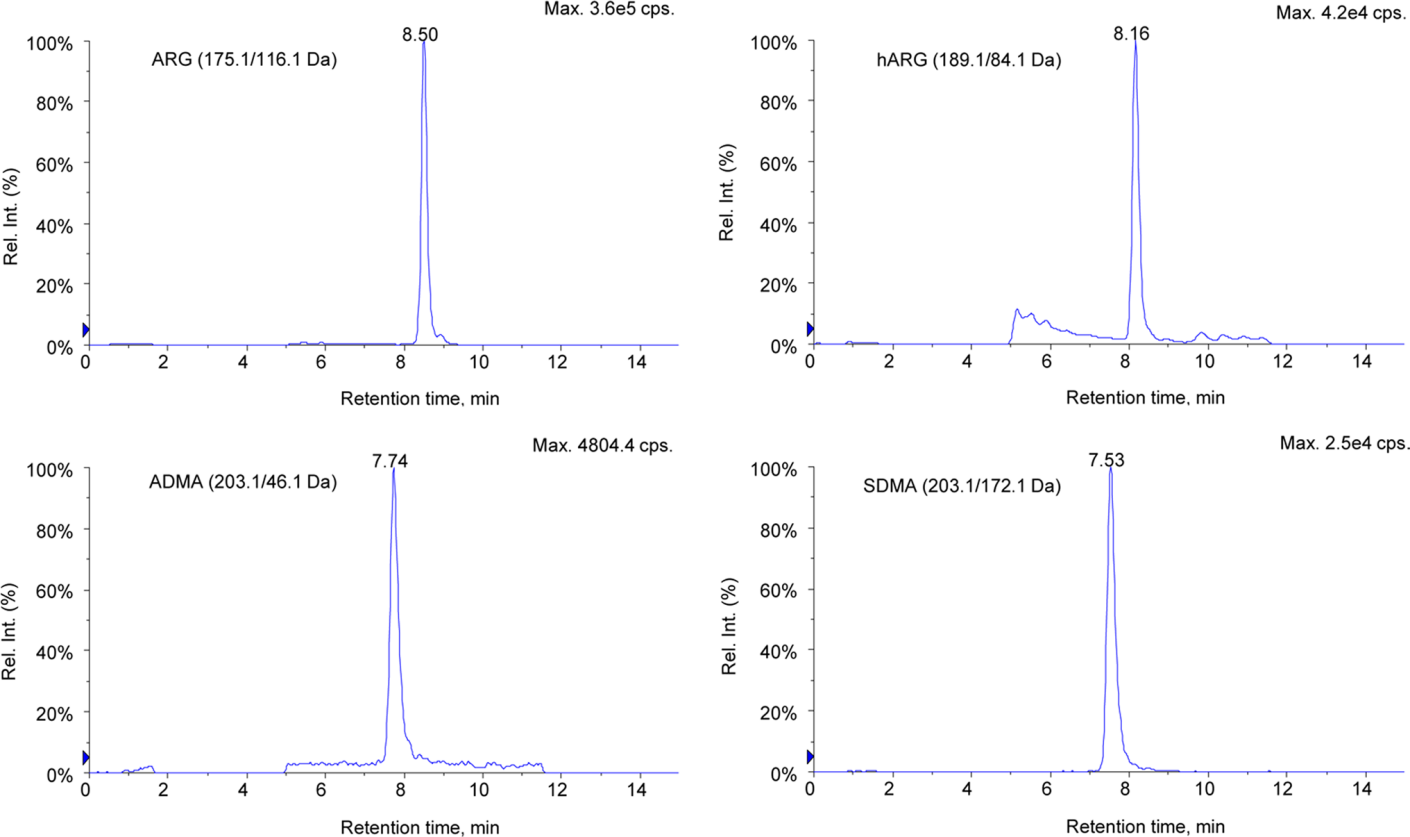
A representative chromatogram showing signals and retention time of four analytes in human serum

ARG, hARG, ADMA, and SDMA could be well distinguished and detected by combining the separation capacity of liquid chromatography with the ability of MS/MS to further select ion pairs for detection (Supplementary Fig. 1, Additional File 1). The chromatographic behavior in serum samples of ARG, hARG, ADMA, and SDMA was shown in Fig. 1, with the retention times of 8.50, 8.16, 7.74, and 7.53 min, respectively.

The linearity of the assay for each analyte was tested in the calibration range by regression analysis. The correlation coefficient r values were > 0.995 for all four analytes (Table 3). The accuracy and precision results were listed in Supplementary Table 1, Additional File 1. The intra-assay and inter-assay accuracy of QC-L, QC-M, and QC-H were within the acceptable standard of 100% ± 15%. Both intra-assay and inter-assay precision met the acceptance criteria that the CV value < 15% among the three levels tested. The extraction recovery range of analytes and IS was 72.4–80.9% and 85.9–89.9% for ARG and ARG-^15^N4, 85.8–95.0% and 85.4–94.3% for hARG and hARG-d4, 93.5–110.0% and 97.5–104.2% for SDMA and SDMA-d6, 92.7–111.5% and 100.4–105.4% for ADMA and ADMA-d6, respectively. The CV of extraction recovery was 1.8–7.0% for analytes and 2.4–6.6% for internal standards, respectively (Supplementary Table 2, Additional File 1). It indicated that the extraction and recovery of analytes and internal standards in serum at different levels were consistent and reproducible.

**Table 3:** Calibration range, linearity, and stability of four analytes in human serum

Analytes	Calibration range, µmol/L	Correlation coefficient, *r* value	Stability, %	
			Room temperature^a^	Freeze–thaw^b^
ARG	10–500	0.99738–0.99959	3.9	− 5.6
hARG	0.2–20	0.99631–0.99875	1.3	2.9
SDMA	0.1–10	0.99888–0.99956	1.1	3.7
ADMA	0.1–5	0.99779–0.99933	− 2.7	− 6.5

^a^Relative deviation of analytes levels in serum samples after storage at room temperature for five hours

^b^Relative deviation of analytes levels in serum samples after three freeze–thaw cycles

The trueness of analytes of different concentrations in different serum samples was within the acceptable criteria of 100% ± 15% (Supplementary Table 3, Additional File 1). The mixing experiment investigated the relative matrix effect. The relative matrix effect ranged from 99.9 to 109.5%, meeting the standard of 80–120%, and CV ranged from 3.7 to 7.9%, which met the standard of CV ≤ 15.0% (Supplementary Table 3, Additional File 1). The results showed that the substitution matrix and serum matrix were consistent in this method. IS-normalized matrix factor ranged from 87.7 to 104.8%, and CV ranged from 1.7 to 6.8%, which met the standard of CV less than 15% (Supplementary Table 4, Additional File 1). The results showed that the internal standard could correct the matrix effect without affecting the accurate quantification of analytes. Four analytes in serum samples after 5 h at room temperature with T0 deviation and after freeze–thaw three times at – 80 ℃with T0 deviation were within 15% (Table 3). Therefore, after rigorous methodological validation, this method was suitable for accurate quantitative detection of ARG, hARG, ADMA, and SDMA concentrations in human serum.

### Difference of serum analytes levels between PE and healthy pregnant women in the same gestational stage

**Fig. 2:**
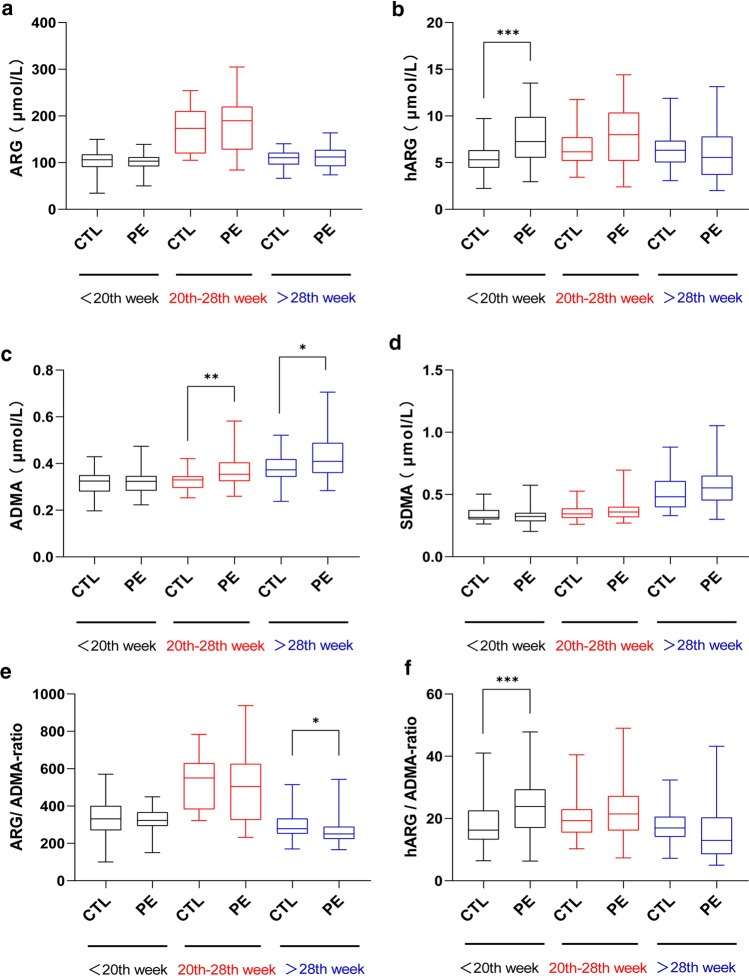
Differences in serum analyte levels between PE patients and controls (CTL) in the same period. Data were expressed from minimum to maximum. *P < 0.05, **P < 0.01, ***P < 0.001, when compared to CTL in the same period

The serum hARG levels and hARG/ADMA ratios of PE before the 20th week were significantly higher than those of controls (*P* < 0.001) (Fig. 2b, 2f). The serum ADMA levels of PE during the 20th-28th week (*P* < 0.01) and after the 28th week (*P* < 0.05) were significantly higher than those of controls in the same period (Fig. 2c). ARG/ADMA ratios were significantly decreased after the 28th week (*P* < 0.05) (Fig. 2e). The serum ARG and SDMA levels of PE were not different from those of controls at any stage (*P* > 0.05) (Fig. 2a, d).

**Fig. 3:**
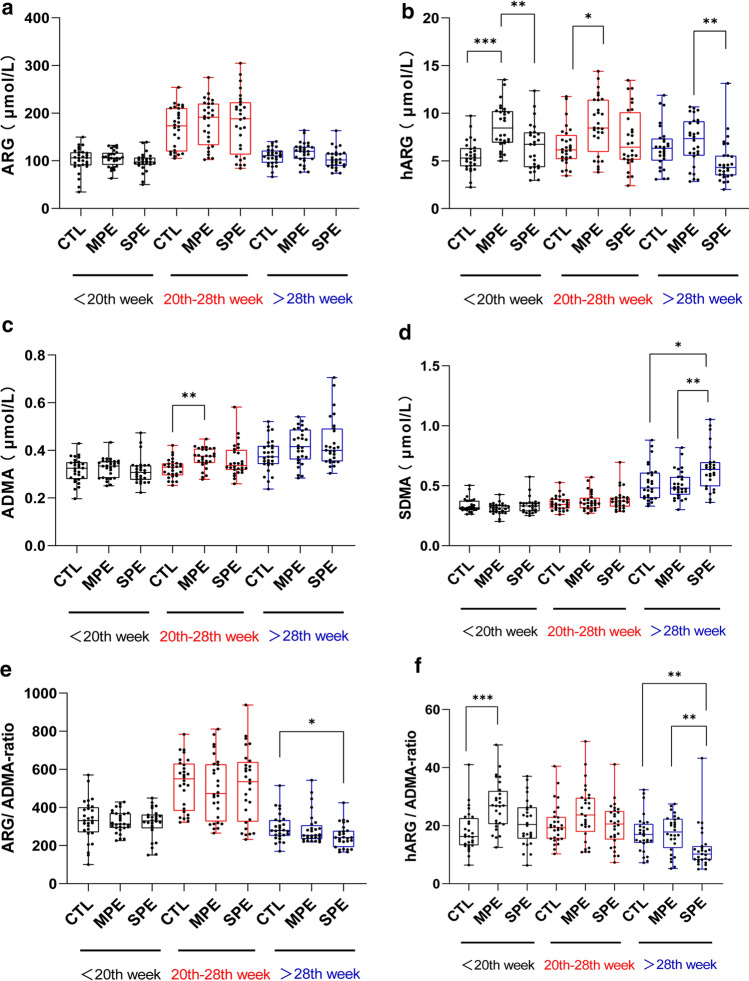
Differences in serum analyte levels among MPE, SPE and controls (CTL) in the same period. Data were expressed from minimum to maximum, showing all points. *P < 0.05, **P < 0.01, ***P < 0.001, when compared with each other in the same period

In the PE subgroup, before the 20th week, the serum hARG levels of MPE were significantly higher than those of controls (*P* < 0.001) and SPE (*P* < 0.01), but there was no significant difference between SPE and controls (*P* > 0.05) (Fig. 3b). The ratios of hARG/ADMA in MPE were also significantly higher than those of controls (*P* < 0.001), but no significant differences of hARG/ADMA were found between MPE and SPE, or SPE and controls (*P* > 0.05) (Fig. 3f). There were no significant differences in serum ARG, ADMA, SDMA levels, and ARG/ADMA ratio among SPE, MPE, and controls before the 20th week (*P* > 0.05) (Fig. 3a, c, d, e).

During the 20th–28th week, the levels of hARG (*P* < 0.05) and ADMA (*P* < 0.01) in MPE were significantly higher than those of controls, but there were no significant differences between SPE and controls (*P* > 0.05) (Fig. 3b, c). There were no significant differences in serum ARG levels, SDMA levels, ARG/ADMA ratio, hARG/ADMA ratio among SPE, MPE, and controls during the 20th–28th week (*P* > 0.05) (Fig. 3a, d, e, f)**.**

After the 28th week, the serum hARG levels of SPE were significantly lower than those of MPE after Bonferroni post-hoc correction (*P* < 0.01), and there was no significant difference between MPE and controls (*P* > 0.05) (Fig. 3b). The serum SDMA levels of SPE were significantly higher than those of MPE (*P* < 0.01) and controls (*P* < 0.05), but there was no significant difference between MPE and controls (*P* > 0.05) (Fig. 3d). The ARG/ADMA ratio of SPE was significantly lower than that of controls (*P* < 0.05), and MPE was not significantly different from SPE and controls (*P* > 0.05) (Fig. 3e). The ratios of hARG/ADMA of SPE were significantly lower than that of MPE (*P* < 0.01) and controls (*P* < 0.01), and there was no significant difference between MPE and controls after the 28th week (*P* > 0.05) (Fig. 3f). There were no significant differences in serum ARG and ADMA levels among SPE, MPE, and controls after the 28th week (*P* > 0.05) (Fig. 3a, c).

To further determine the factors associated with PE before the 20th week of gestation, the variables with *P* < 0.2 from the univariate analysis were entered into the multivariate logistic regression analysis with a forward stepwise method. Serum hARG levels (*P* = 0.006) and primiparity (*P* = 0.027) were identified as the independent predictors of PE before the 20th week of gestation (Table 4).

**Table 4:** Result of logistic regression analysis before the 20th week

Variable	Univariate analysis	Multivariate logistic regression analysis	
	*P *value	Odds ratio (95CI%)	*P* value
Serum hARG level	< 0.001	1.478 (1.120–1.950)	0.006
hARG/ADMA ratio	< 0.001	/	0.691
Primiparity	0.002	3.403 (1.148–10.084)	0.027
Age	0.061	/	0.499
GFR	0.121	/	0.497

*GFR* glomerular filtration rate

### Predictive ability of serum hARG, ARG, SDMA and ADMA for PE

**Fig. 4:**
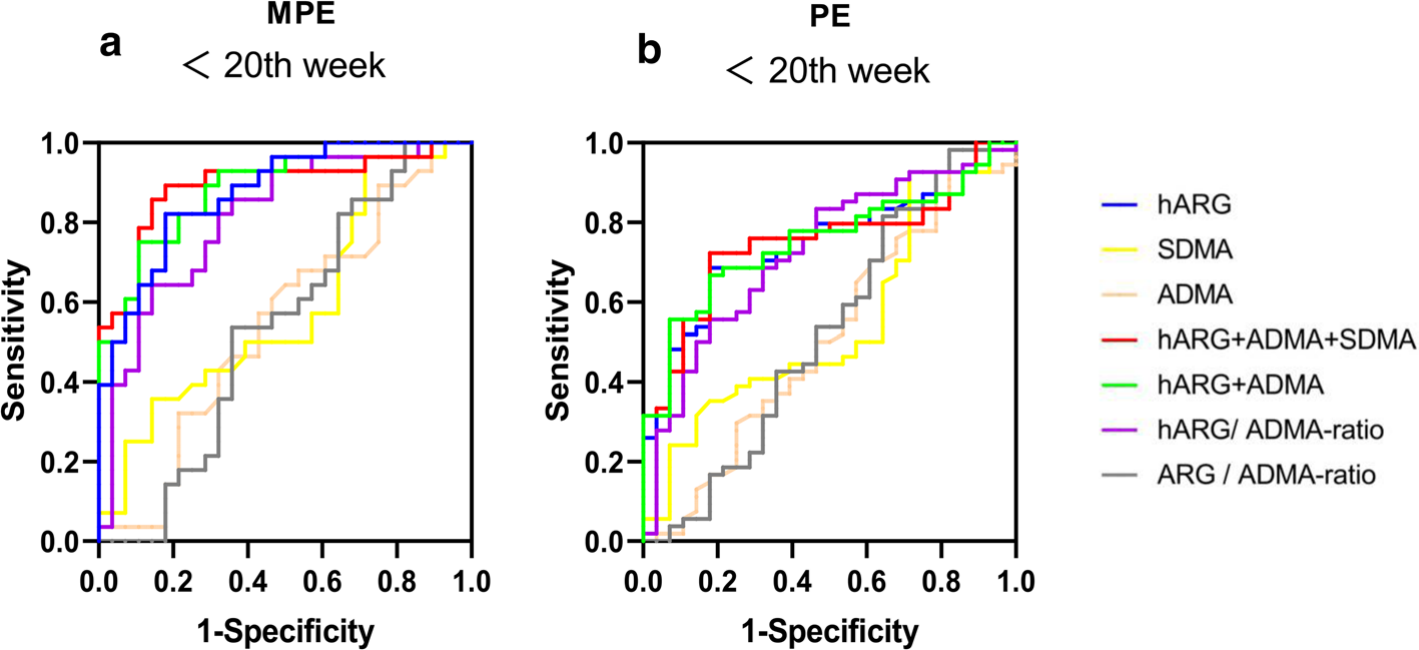
ROC curve analysis of the single serum analyte and combination panels predicting MPE **a** and all PE **b** before the 20th week. hARG + ADMA represents the combination panels of the serum hARG and ADMA levels. hARG + ADMA + SDMA represents the combination panels of the serum hARG, ADMA and SDMA levels

Before the 20th week of pregnancy, the AUCs of serum hARG, hARG/ADMA ratio, and the combination panels (hARG + ADMA and hARG + ADMA + SDMA) for predicting PE or MPE were significantly higher than those of the other serum analytes (*P* < 0.05) (Fig. 4a, b and Supplementary Table 5). The AUC of serum hARG to predict MPE or PE was 0.875 (95% CI 0.759–0.948) or 0.746 (95% CI 0.638–0.836). The AUC of hARG + ADMA combination for predicting MPE or PE was 0.890 (95% CI 0.778–0.958) or 0.751 (95% CI 0.644 –0.840). The AUC of hARG + ADMA + SDMA combination for predicting MPE or PE was 0.893 (95% CI 0.781–0.960) or 0.749 (95% CI 0.641–0.838). There was no significant difference in AUCs between serum hARG and the combination panels (hARG + ADMA and hARG + ADMA + SDMA) before the 20th week when they were used to predict MPE or PE (*P* > 0.05). Although the AUC of hARG/ADMA ratio (0.810, 95% CI: 0.683–0.902) was significantly lower than that of serum hARG for predicting MPE (0.875, 95% CI 0.759–0.948), it was significantly higher than that of ARG/ADMA ratio before the 20th week for predicting MPE (0.538, 95% CI 0.400–0.672, *P* < 0.05).

During the 20th and 28th week of gestation, the AUCs of serum ADMA and the combination panels for predicting MPE or PE were significantly higher than those of the other serum analytes (*P* < 0.05). There was no significant difference in AUCs between serum ADMA and the combination panels (*P* > 0.05) (Supplementary Table 5). After the 28th week of gestation, the AUCs of the single analyte and the combination panels for predicting MPE or PE were all less than 0.7.

## Discussion

In this study, a stable isotope dilution LC–MS/MS method was established to accurately detect the levels of serum hARG, ARG, ADMA, and SDMA. Only 50 µL serum was needed to simultaneously detect the levels of serum hARG, ARG, ADMA, and SDMA. After rigorous methodological verification, the results were considered stable, accurate, and repeatable. There was no significant difference in serum ARG levels between PE and the normal pregnant women at the same gestation stage, consistent with what Mao D and his colleagues found (Mao et al. 2010). There was also no significant difference in serum ADMA levels between PE and normal pregnant women before the 20th week, consistent with Khalil and colleagues’ findings (Khalil et al. 2013). However, we found serum ADMA levels of PE after the 20th week were significantly higher than those of a normal pregnancy, which was consistent with the findings of Selanno and Savvidou (Selanno et al. 2020; Savvidou et al. 2003). We found comparable outcomes when the subgroups were divided into trimesters (Supplemental Table 6, Additional File 1). The ARG/ADMA ratios of SPE were significantly lower than those of the normal pregnant after the 28th week, as found by Tashie W and colleagues (Tashie et al. 2020). It suggested a decline in NO synthesis and severe impairment of endothelial function in SPE after the 28th week. When ARG and ADMA were at physiological concentrations, NOS was completely saturated by ARG. However, when ADMA increased pathologically, it competitively inhibited the activity of NOS, leading to a decrease in NO synthesis. Studies showed that exogenous ARG supplements play an essential role in enhancing NO synthesis (Camarena Pulido et al. 2016; Weckman et al. 2019). Serum SDMA levels in SPE after the 28th week were significantly higher than those in the other groups, consistent with Ellis J and colleagues (Ellis et al. 2001). SDMA was an emerging marker for assessing early renal function, and elevated serum SDMA levels might be associated with the general impairment of renal function in SPE in the third trimester (Schwedhelm and Boeger 2011). We speculated that serum SDMA levels after the 28th week of gestation might be a more sensitive indicator for monitoring renal impairment than GFR.

Compared with the relationships between PE and ARG, NO, or ADMA, the relationship between PE and hARG was not fully revealed. This study found the differences in serum hARG between PE and normal pregnant women at different gestational weeks. In this study, serum hARG levels and hARG/ADMA ratio of SPE decreased significantly after the 28th week. The serum hARG levels of SPE were significantly lower than those of MPE after the 28th week. It may be related to PE patients’ severely impaired endothelial function, which causes severe hypertension or terminal organ damage. In some populations, low serum hARG has recently emerged as a potential new risk marker for cardiovascular mortality (Atzler et al. 2014). Low hARG levels may be an early indicator of renal failure and a potential target to prevent disease progression (Drechsler et al. 2013). The decrease of hARG level may increase TNAP (tissue-nonspecific alkaline phosphatase) activity (Kozlenkov et al. 2004; Karetnikova et al. 2019). TNAP is expressed in the kidney, bone, liver, and vascular endothelial cells. Epidemiological studies have shown that the increased concentration of TNAP in the blood is an independent predictor of the total mortality of patients with coronary artery disease (Park et al. 2013). Whether low serum hARG has a biological effect on pregnancy through TNAP, or it is only a non-causal sign of adverse effects of preeclampsia, it needs to be further studied in the future.

The serum hARG levels of MPE were found significantly higher than those of normal pregnancy before the 28th week. The elevated serum hARG levels occurred before MPE diagnosis in this study. Some studies suggested that for abnormal pregnancies such as IUGR and PE, hARG was a vasodilator to address these abnormalities (Valtonen et al. 2008; Adams et al. 2019). We speculated that elevated hARG might be related to vascular maladaptations during the occurrence and development of MPE (Hedman et al. 2020). This might be a compensatory mechanism in response to endothelial dysfunction. hARG can be obtained from food or synthesized from lysine and arginine catalyzed by arginine glycine amidino transferase (AGAT) (Kayacelebi et al. 2015; Papageorgiou et al. 2015). The way to produce hARG through the urea cycle in vivo is still uncertain (Davids et al. 2012; Choe et al. 2013). In addition to being decomposed into NO and homocitrulline by NOS, hARG can also be decomposed into lysine and urea by arginase. It can also be decomposed into 6-guanidino-2-oxocaproic acid (GOCA) by alanine glyoxylate aminotransferase 2 (AGXT2) (Karetnikova et al. 2019; Rodionov et al. 2016). Two large genome-wide association studies revealed that the AGAT gene's single nucleotide polymorphisms (SNPs) were related to plasma hARG concentrations (Choe et al. 2013; Kleber et al. 2013). More researches are needed to explore the role of hARG anabolic abnormality and downstream signaling in MPE vascular maladaptation. Additionally, the circulating levels of ARG are approximately 30 times higher than the circulating levels of hARG, and the Km of NOS to hARG is ten times higher than that of ARG (Moali et al. 1998; Marz et al. 2010). It is doubtful that hARG can directly improve NO’s bioavailability through NOS to exert endothelial protection. Furthermore, some studies suggested the effect of hARG on vascular endothelium may be achieved by antagonizing NG-methylated protein, a precursor protein that hydrolyzes and releases free ADMA and SDMA (Tsikas et al. 2018). Although MPE patients in this study had an increased proportion of primiparity, our logistic regression analysis indicated that serum hARG level before the 20th week was an independent predictor for PE. However, whether elevated hARG was an independent risk factor for PE, or just a compensatory outcome to correct abnormal pregnancy in the early stage of PE, it requires further research in the future. Logistic regression analysis also showed that primiparity was an independent risk factor for PE, which was consistent with previous findings (Duckitt and Harrington 2005).

Our ROC curve analysis found that before the 28th week of gestation, neither single analyte nor combination panels were good at predicting SPE (AUC: 0.510–0.636, data not shown). It may be related to the complex pathogenesis of SPE involving multiple pathophysiologic factors, diverse clinical symptoms, and individual differences in SPE. ROC curve analysis also indicated that serum hARG levels had a good potential to predict MPE before the 20th week of gestation, with an AUC of 0.875. Moreover, the predictive performances of the combination panels for MPE were not better than that of the single serum hARG at the 20th week of gestation. MPE may progress to SPE rapidly, although MPE has no obvious clinical symptoms at the beginning of the disease and cannot be diagnosed and differentiated until clinical symptoms occurred (Hennessy and Makris 2011). According to the American College of Obstetricians and Gynecologists (ACOG) in 2013, even if MPE does not have severe clinical manifestations, it may deteriorate within a short period, leading to serious life-threatening complications. Intensive monitoring is required (ACOG Practice Bulletin No 2019). Therefore, early prediction of MPE, early assessment of maternal risk before the onset of clinical symptoms, and early management are also indispensable to reduce mortality associated with MPE. The serologic differences between MPE and SPE suggested a different developmental mechanism, which needs further study in the future.

Although the ARG/ADMA ratio has been proposed as an indicator of NO, the role of the hARG/ADMA ratio in disease has not been revealed. This study found that compared with the ARG/ADMA ratio, the hARG/ADMA ratio might be an earlier and more sensitive biomarker in PE. The AUC of hARG /ADMA ratio was significantly higher than that of ARG/ADMA ratio for predicting MPE before the 20th week. Compared with normal pregnancy, the hARG/ADMA ratio of MPE increased significantly before the 20th week, while the hARG/ADMA ratio of SPE decreased after the 28th week. However, there was no difference between the ARG/ADMA ratio of PE and that normal pregnancy before the 28th week. It has been reported that short-term hARG supplementation has beneficial effects on mouse stroke models (Choe et al. 2013). We speculate that in the case of elevated serum ADMA levels in SPE, exogenous hARG supplementation may be more effective than ARG in reducing the clinical severity of SPE.

In summary, we developed and validated a practicable clinical assay to separate and quantify serum ARG, hARG, ADMA, SDMA levels by LC–MS/MS. Using this method, we detected the levels of these analytes in the serum and revealed their differences in different stages of pregnancy with or without PE. Our study indicated that elevated serum hARG and dimethylarginine levels detected by LC–MS/MS might serve as potential biomarkers for the early prediction of PE. Maternal serum hARG levels and ADMA levels were elevated in PE patients before and after the 20th week of gestation, respectively. Elevated serum hARG before the 20th week was identified as an independent predictor for PE. In addition, we observed a significant increase in serum SDMA levels in patients with SPE after 28 weeks of gestation. Due to the small cohort of subjects, our clinical findings were preliminary. Large-scale studies are needed to verify the clinical predictive value of hARG in PE and to elucidate the underlying pathophysiological mechanism of hARG involved in pregnancy at different stages.

## Supplementary Information

Below is the link to the electronic supplementary material.

Supplementary file1 (PDF 451 KB)

## Data Availability

The datasets used or analyzed during the current study are available from the corresponding author on reasonable request.
