# Supplementary material for: Evaluation of the predictive values of elevated serum l-homoarginine and dimethylarginines in preeclampsia
Source: Amino Acids. 2022 Jun 26;54(8):1215–27. doi: 10.1007/s00726-022-03177-x (PMC9365731; doi:10.1007/s00726-022-03177-x)
Supplement: Supplementary file 1 — Supplementary file1 (PDF 451 KB) [file 726_2022_3177_MOESM1_ESM.pdf]

# Evaluation of the predictive values of elevated serum L-homoarginine and dimethylarginines in preeclampsia

Xiangmei Yuan[1†], Leiming Cai[1†], Fengmei Hu[2], Li Xie[1], Xiong Chen[3], Jingjing Wu[3], Qian Li[1*]

[*] Correspondence: liqian@ws-hospital.sh.cn

[†]Xiangmei Yuan and Leiming Cai contributed equally to this work

[1]Department of Laboratory Medicine, Wusong Branch, Zhongshan Hospital, Fudan University, Shanghai 200940, China.

[2]Shanghai AB Sciex Analytical Instrument Trading Co., Ltd., Shanghai 200050, China.

[3]Department of Gynecology and obstetrics, Wusong Branch, Zhongshan Hospital, Fudan University, Shanghai 200940, China.

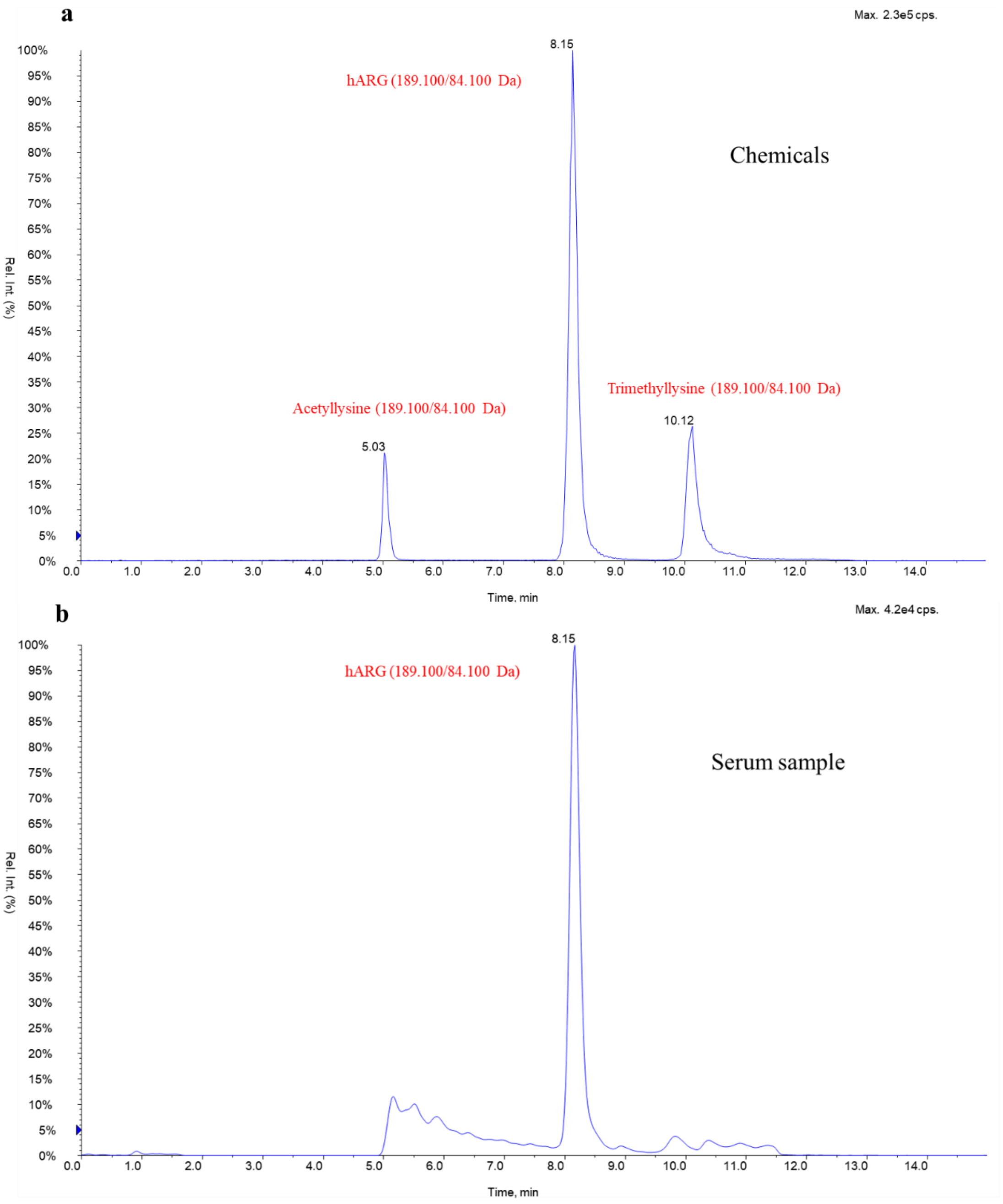


**Supplemental Figure 1** Chromatographic behavior of hARG, trimethyllysine and acetyllysine in chemicals (a) and serum sample (b)

**Supplementary Table 1** The accuracy and precision in method validation.

| Analyte | Spiked concentration, µmol/L | Accuracy, % | | Precision CV, % | |
|---|---|---|---|---|---|
| | | Intra-assay | Inter-assay | Intra-assay | Inter-assay |
| ARG | QC-L | 91.2 | 96.4 | 2.3 | 5.7 |
| | | 101.6 | | 7.1 | |
| | | 96.4 | | 6.1 | |
| | QC-M | 95.4 | 99.0 | 4.1 | 5.1 |
| | | 96.0 | | 3.0 | |
| | | 105.7 | | 1.7 | |
| | QC-H | 97.1 | 96.2 | 2.3 | 4.4 |
| | | 95.1 | | 6.2 | |
| | | 96.2 | | 4.2 | |
| hARG | QC-L | 100.8 | 98.5 | 8.5 | 5.9 |
| | | 98.0 | | 5.1 | |
| | | 96.6 | | 1.4 | |
| | QC-M | 101.8 | 101.3 | 6.5 | 3.7 |
| | | 101.4 | | 1.7 | |
| | | 100.7 | | 4.1 | |
| | QC-H | 97.2 | 99.4 | 6.0 | 4.9 |
| | | 98.5 | | 1.8 | |
| | | 102.5 | | 5.3 | |
| SDMA | QC-L | 99.4 | 96.8 | 4.9 | 5.3 |
| | | 94.1 | | 4.3 | |
| | | 96.8 | | 7.3 | |
| | QC-M | 102.0 | 102.3 | 2.7 | 1.7 |
| | | 102.6 | | 1.7 | |
| | | 102.4 | | 1.8 | |

| Analyte | Spiked concentration, µmol/L | Accuracy, % | | Precision CV, % | |
|---|---|---|---|---|---|
| | | Intra-assay | Inter-assay | Intra-assay | Inter-assay |
| | QC-H | 90.7 | 91.5 | 4.3 | 4.3 |
| | | 92.1 | | 4.2 | |
| | | 91.9 | | 7.8 | |
| ADMA | QC-L | 98.4 | 99.1 | 3.8 | 4.4 |
| | | 100.9 | | 1.9 | |
| | | 100.6 | | 3.7 | |
| | QC-M | 104.2 | 102.6 | 7.4 | 4.5 |
| | | 101.4 | | 1.6 | |
| | | 102.2 | | 2.6 | |
| | QC-H | 101.0 | 101.0 | 3.4 | 5.3 |
| | | 104.9 | | 4.6 | |
| | | 97.3 | | 5.3 | |

**Supplementary Table 2** The extraction recovery of analyte and IS in method validation.

| Analyte | Low level | | High level | | IS | Low level | | High level | |
|---|---|---|---|---|---|---|---|---|---|
| | Recovery, % | CV, % | Recovery, % | CV, % | | Recovery, % | CV, % | Recovery, % | CV, % |
| ARG | 72.4 | 3.2 | 80.9 | 2.2 | ARG-$^{15}N_4$ | 85.5 | 3.5 | 89.9 | 3.6 |
| hARG | 85.8 | 6.3 | 95.0 | 7.0 | hARG-d4 | 85.4 | 5.6 | 94.3 | 2.9 |
| SDMA | 110.0 | 4.4 | 93.5 | 5.4 | SDMA-d6 | 97.5 | 2.4 | 104.2 | 3.5 |
| ADMA | 111.5 | 5.9 | 92.7 | 1.8 | ADMA-d6 | 100.4 | 2.4 | 105.4 | 6.6 |

**Supplementary Table 3** The trueness and relative matrix effect in method validation.

| Analyte | Trueness, % | | CV, % | | Relative matrix effect, % | | CV, % | |
|---|---|---|---|---|---|---|---|---|
| | Low | High | Low | High | Low | High | Low | High |
| ARG | 96.2 | 90.3 | 6.6 | 4.4 | 101.1 | 101.4 | 6.2 | 5.0 |
| hARG | 93.7 | 90.2 | 4.4 | 3.8 | 103.0 | 103.5 | 7.0 | 6.4 |
| SDMA | 97.7 | 93.0 | 7.7 | 5.7 | 105.1 | 99.9 | 7.9 | 3.7 |
| ADMA | 93.5 | 89.7 | 4.0 | 5.0 | 109.5 | 103.2 | 5.5 | 5.8 |

**Supplementary Table 4** The absolute matrix effect and IS-normalized matrix factor in method validation.

| Analyte | Absolute matrix effect, % | CV, % | IS-normalized matrix factor, % | CV, % |
|---|---|---|---|---|
| ARG | 91.9 | 1.1 | 101.9 | 4.4 |
| | 93.4 | | 93.3 | |
| | 91.4 | | 97.0 | |
| hARG | 98.5 | 8.5 | 104.2 | 6.5 |
| | 92.5 | | 93.2 | |
| | 109.3 | | 104.8 | |
| SDMA | 94.7 | 8.5 | 104.2 | 1.7 |
| | 100.9 | | 93.2 | |
| | 97.8 | | 104.8 | |
| ADMA | 105.1 | 4.1 | 99.0 | 6.8 |
| | 100.6 | | 87.7 | |
| | 109.4 | | 98.8 | |

**Supplementary Table 5** Predictive ability of single analyte or combination panels to predict MPE or PE before the 28th week

| **Predictor** | **Group** | **AUC** | **95%CI** | **Cut-off value (μmol/L)** | **Sensitivity (%)** | **Specificity (%)** |
|---|---|---|---|---|---|---|
| **Before the 20th week** | | | | | | |
| hARG | MPE | 0.875 | 0.759 - 0.948 | 6.72 | 82.1 | 82.1 |
| | PE | 0.746 | 0.638- 0.836 | 6.68 | 68.5 | 82.1 |
| hARG/ADMA-ratio | MPE | 0.810 | 0.683 - 0.902 | 18.48 | 85.7 | 64.3 |
| | PE | 0.726 | 0.616- 0.818 | 23.12 | 55.6 | 82.1 |
| hARG + ADMA | MPE | 0.890 | 0.778 - 0.958 | / | / | / |
| | PE | 0.751 | 0.644- 0.840 | / | / | / |
| hARG+ADMA+SDMA | MPE | 0.893 | 0.781 - 0.960 | / | / | / |
| | PE | 0.749 | 0.641 - 0.838 | / | / | / |
| ADMA | MPE | 0.549 | 0.410 - 0.682 | / | / | / |
| | PE | 0.509 | 0.396 - 0.621 | / | / | / |
| SDMA | MPE | 0.586 | 0.447 - 0.716 | / | / | / |
| | PE | 0.549 | 0.435 - 0.659 | / | / | / |
| ARG/ADMA-ratio | MPE | 0.538 | 0.400 - 0.672 | / | / | / |
| | PE | 0.525 | 0.412 - 0.637 | / | / | / |
| **During the 20th–28th week** | | | | | | |
| hARG | MPE | 0.714 | 0.578 - 0.827 | 7.33 | 75.0 | 75.0 |

| | | | | | | |
|---|---|---|---|---|---|---|
| | PE | 0.625 | 0.512 - 0.728 | 7.33 | 58.9 | 75.0 |
| ADMA | MPE | 0.784 | 0.653 - 0.883 | 0.345 | 78.6 | 75.0 |
| | PE | 0.710 | 0.601 - 0.804 | 0.345 | 60.7 | 75.0 |
| hARG/ADMA-ratio | MPE | 0.631 | 0.492 - 0.756 | 20.23 | 67.9 | 64.3 |
| | PE | 0.575 | 0.463 - 0.682 | 23.08 | 46.4 | 78.6 |
| hARG + ADMA | MPE | 0.849 | 0.729 - 0.931 | / | / | / |
| | PE | 0.728 | 0.620 - 0.819 | / | / | / |
| hARG+ADMA+SDMA | MPE | 0.869 | 0.752 - 0.944 | / | / | / |
| | PE | 0.739 | 0.631 - 0.828 | / | / | / |
| SDMA | MPE | 0.555 | 0.416 - 0.688 | / | / | / |
| | PE | 0.579 | 0.466 - 0.686 | / | / | / |
| ARG/ADMA-ratio | MPE | 0.563 | 0.423 - 0.695 | / | / | / |
| | PE | 0.547 | 0.435 - 0.656 | / | / | / |

**Supplementary Table 6** Serum analyte levels in the first, second and third trimesters

| **Analytes** | **CTL** | **MPE** | **SPE** |
|---|---|---|---|
| **The first trimester** | | | |
| Number | N=17 | N=13 | N=15 |
| ARG (μmol/L) | 111.98 ± 16.61 | 104.24 ± 18.89 | 95.60 (92.15 – 103.00) |
| hARG (μmol/L) | 5.40 (4.96 - 6.63) | 7.27 (6.87 – 10.55) *** | 6.27 ± 2.54 |
| ADMA (μmol/L) | 0.319 ± 0.059 | 0.347 (0.283 – 0.354) | 0.307 ± 0.044 |
| SDMA (μmol/L) | 0.314 (0.298 – 0.376) | 0.313 ± 0.044 | 0.325 ± 0.054 |

| | | | |
|---|---|---|---|
| **The second trimester** | | | |
| Number | N=39 | N=43 | N=39 |
| ARG (µmol/L) | 133.80 (108.24 – 192.70) | 132.40 (107.15 – 202.90) | 132.50 (100.22 – 210.10) |
| hARG (µmol/L) | 6.07 ± 2.11 | 8.67 ± 2.69 *** | 7.13 ± 2.88 |
| ADMA (µmol/L) | 0.321 ± 0.039 | 0.354 ± 0.048 ** | 0.329 (0.305– 0.391) |
| SDMA (µmol/L) | 0.339 (0.307 – 0.379) | 0.334 (0.303– 0.372) | 0.358 (0.320– 0.401) |
| **The third trimester** | | | |
| Number | N=28 | N=28 | N=27 |
| ARG (µmol/L) | 109.09 ± 18.00 | 116.88 ± 21.81 | 103.97 ± 20.40 |
| hARG (µmol/L) | 6.44 ± 2.29 | 7.02 ± 2.47 | 4.31 (3.48, 5.56) * |
| ADMA (µmol/L) | 0.380 ± 0.069 | 0.418 ± 0.074 | 0.400 (0.354, 0.492) |
| SDMA (µmol/L) | 0.482 (0.397, 0.609) | 0.508 ± 0.119 | 0.637 (0.495, 0.697) * |

Values are expressed as median (IQR 25 - 75%) or mean ± standard variation. *$P < 0.05$, **$P < 0.01$, ***$P < 0.001$, when compared to CTL in the same period.
